# Long-term Histopathologic Follow-up of a Spherical Embolic Agent; Observation of the Transvascular Migration of HepaSphere^TM^

**DOI:** 10.1259/bjrcr.20180066

**Published:** 2019-01-25

**Authors:** Norifumi Kennoki, Toru Saguchi, Toru Sano, Yuki Takara, Tomohisa Moriya, Natsuhiko Shirota, Jun Otaka, Naokazu Chiba, Shigeyuki Kawachi, Hiromi Serizawa, Kiyoshi Koizumi, Koichi Tokuuye

**Affiliations:** 1 Department of Radiology, Tokyo Medical University Hospital, Tokyo, Japan; 2 Department of Digestive and Transplantation Surgery, Tokyo Medical University Hachioji Medical Center, Tokyo, Japan; 3 Department of Radiology, Tokyo Medical University Hachioji Medical Center, Tokyo, Japan; 4 Department of Diagnostic Pathology, Tokyo Medical University Hachioji Medical Center, Tokyo, Japan

## Abstract

Very few studies have been published on the long-term histopathologic follow-up of spherical embolic agents after their injection. To our knowledge, there are no reports in the literature regarding pathological analysis of the transvascular migration of HepaSphere particles. We here report a case of a patient with hepatocellular carcinoma (HCC) who underwent liver transplantation 12 months after drug eluting microsphere transcatheter arterial chemoembolization (DEM-TACE), and long-term histopathologic follow-up of the microspheres was performed. Furthermore, to our knowledge, this is the first report in which transvascular migration of a HepaSphere particle was confirmed histologically. A 60-year-old male with chronic hepatitis B was treated with entecavir and seroconversion was obtained. The patient had decompensated cirrhosis, and desired to undergo living donor liver transplantation (LDLT). However, 2 HCC tumors of 3  cm or less were detected in his liver. The transplantation surgeon proposed DEM-TACE as a bridge therapy. The HCCs were located in the right lobe and lateral segment of the liver. A 1.9  F preshaped microcatheter (ProgreatΣ, Terumo, Japan) was selectively inserted into the A3 and anterior segmental branch, 10  mg of epirubicin was injected into each artery, and the arteries were embolized with 7  mg and 13  mg of HepaSphere loaded with epirubicin, respectively. Two months later, contrast-enhanced CT displayed a complete response. At that time, lung metastasis was suspected, but after partial lung resection, the patient was diagnosed as having inflammatory granuloma. One year after DEM-TACE treatment, LDLT was performed. No cancerous cells were detected in the area where the tumor was present, but 22 HepaSphere particles were detected. All particles were present in the interstitium. Furthermore, the transvascular migration of a HepaSphere particle was histologically confirmed. The largest and smallest HepaSphere diameters were 241.6  ±  52.5  µm and 186.5  ±  41.4  µm, respectively, and deformity was 22.6% ± 13.0 %. All the HepaSpheres detected in the examined pathological specimen were noted to be extravascular.

## Introduction

There have been very few reports on pathological long-term follow-up after embolization surgery using spherical embolic material. The possibility that HepaSphere, a permanent embolic material, leaks out of the blood vessels after embolization has been suggested,^1^ which is still under debate and the mechanism is still unknown. In the present case, drug-eluting microsphere transcatheter arterial chemoembolization (DEM – TACE) was performed as a bridging therapy before liver transplantation, and pathological analysis was performed 12 months after the embolization. To our knowledge, this is the first report of the immunohistological confirmation of the transvascular migration of HepaSphere particles.

## Case presentation

This case was exempted from ethical approval by the hospital ethics committee. Written informed consent for the case to be published (incl. images, case history and data) was obtained from the patient for publication of this case report, including accompanying images. A 60-year-old male with chronic hepatitis B was treated with entecavir and seroconversion was confirmed. His Child–Pugh score was B, platelet count was 110,000, and the patient wished to undergo living donor liver transplantation (LDLT) for his decompensated cirrhosis. However, 2 hepatocellular carcinoma (HCC) tumors of 3 cm or less were detected in his liver, and the transplantation surgeon proposed DEM-TACE as a bridge therapy before LDLT to prevent from tumor growth and increase.

## Procedure

The procedure of DEM-TACE adopted the report of Hori et al.^2^ HepaSphere (50–100 µm) loaded with 20 mg of epirubicin was used as embolic material. The reduced expansion technique was used;^3^ *i.e.* 20 mg of epirubicin powder was dissolved in a 5 ml solution of 1 ml of 10% NaCl and 4 ml of nonionic contrast material and loaded with a vial (25 mg) of dry HepaSphere.

**Figure 1.**
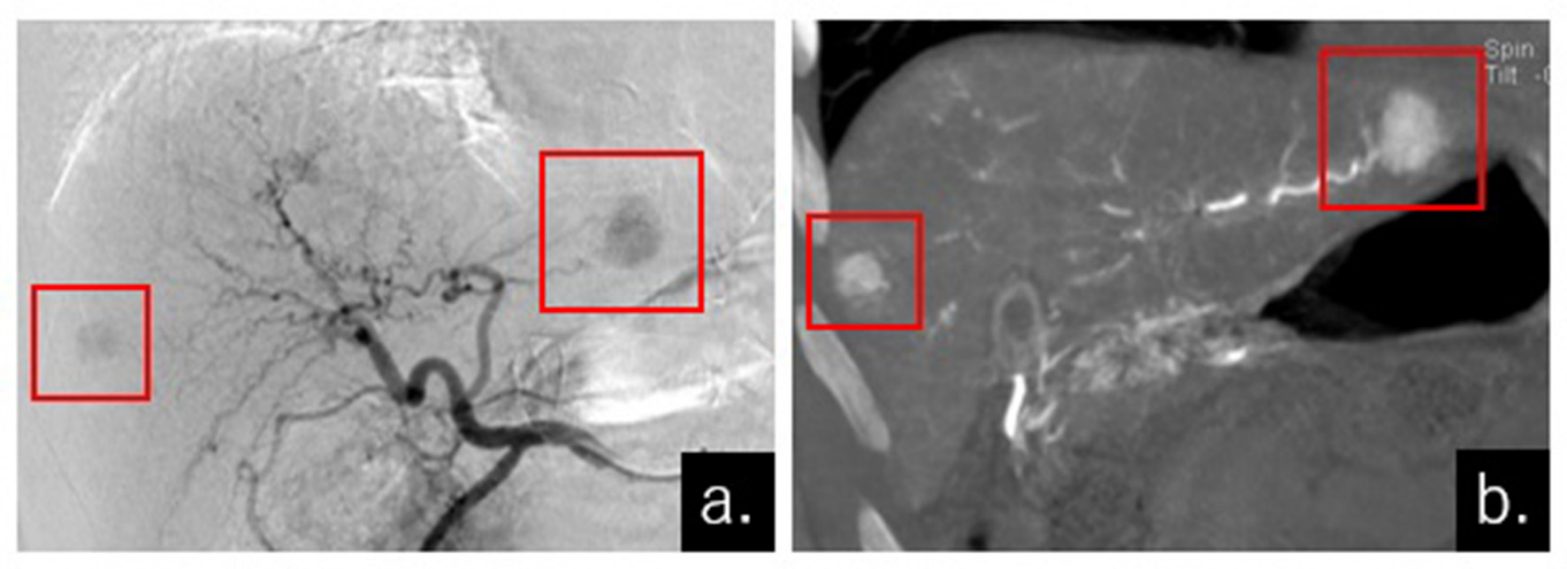
DSA (digital subtraction angiography) and CTA (CT angiography) performed during the DEM-TACE procedure. The right femoral artery was punctured and a 4 F long sheath and guiding catheter was advanced coaxially into the common hepatic artery (CHA). Digital subtraction angiography (DSA, (a) and CT angiography (CTA, (b) were performed from the CHA and 2 tumors were stained, in the right lobe and in the lateral segment of the liver (red squares).

**Figure 2.**
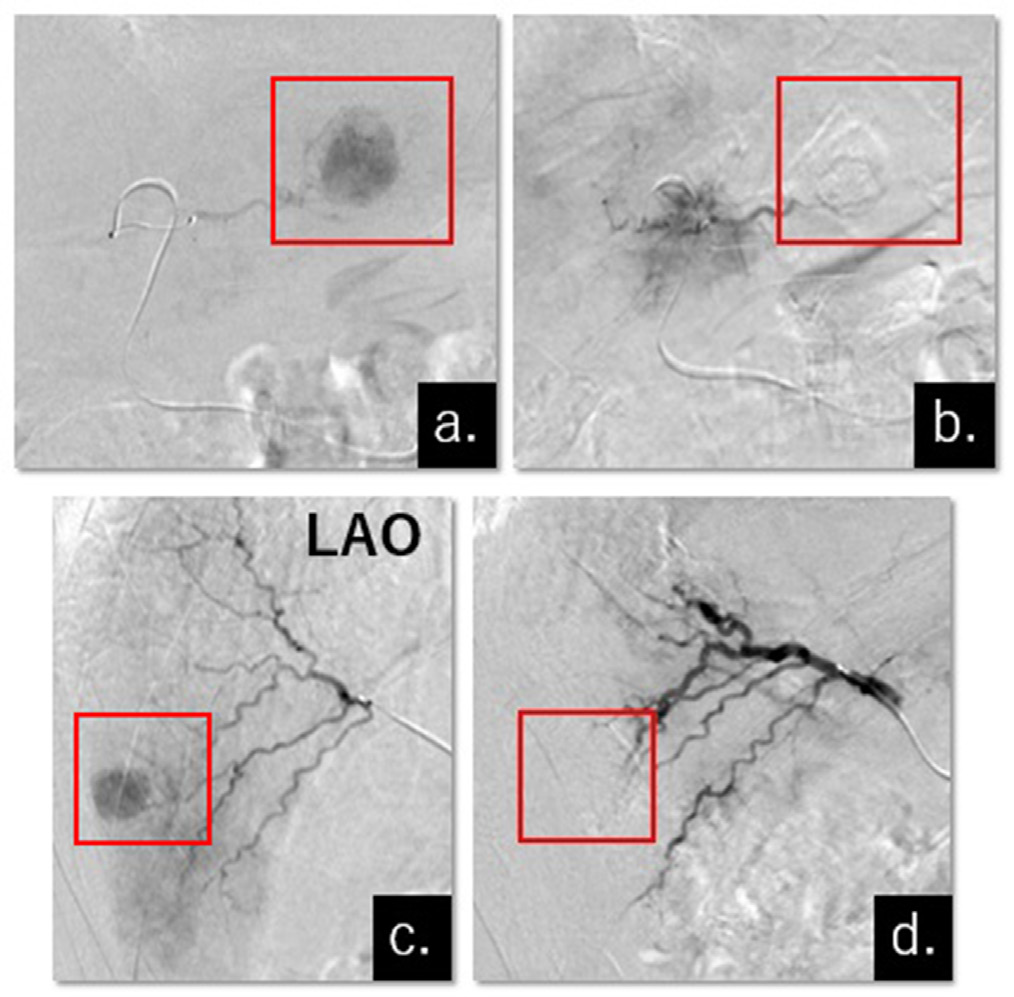
DSA performed before and after embolization with HepaSphere (a) The microcatheter was super-selectively advanced into the A3, and tumor staining was displayed in S3 (red square).(b) Tumor staining in S3 completely disappeared after the embolization (red square).(c) DSA was performed from the anterior segmental branch at the angle of the lateral anterior oblique, and tumor staining in S5 was displayed (red square).(d).Tumor staining in S5 completely disappeared after the embolization (square).

The right femoral artery was punctured under local anesthesia and a 4 F long sheath was inserted. Next, a 4 F guiding catheter (MM1, Gadelius Medical K.K., Japan) was advanced into the common hepatic artery. Digital subtraction angiography (DSA) and CT angiography were performed from the artery and 2 tumor stains were displayed in the right lobe and lateral segment of the liver (Figure 1). The size of the S3 and 5 tumors were 12 × 12 × 10 mm and 20 × 18 × 14 mm, respectively. A 1.9 F preshaped microcatheter (ProgreatΣ, Terumo, Japan) was selectively inserted into the A3 and anterior segmental branch and tumor staining was confirmed on DSA (Figure 2a,c). Ten mg of epirubicin solution was injected from each artery. Immediately after infusion of epirubicin, 0.5–1.0 mg of HepaSphere was mixed with 1 ml of nonionic contrast material and injected under fluoroscopy very slowly (1 ml sec^–1^) using a 1 ml syringe, until the tumor staining disappeared completely (Figure 2b,d). The total dose of HepaSphere administered was 20 mg; 7 mg for A3 and 13 mg for the anterior segmental branch, respectively.

## Follow up

**Figure 3.**
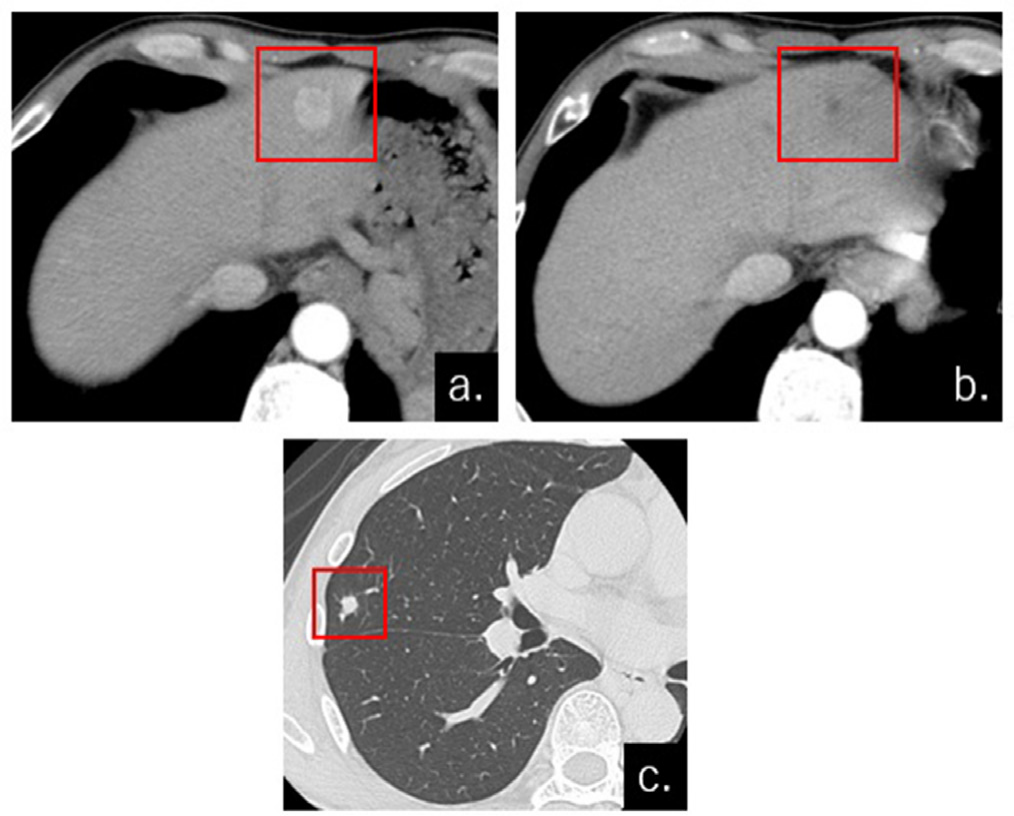
Contrast-enhanced CT (CECT) before and 2 months after embolization with HepaSphere (a) CECT before the procedure demonstrated HCC tumor staining in S3, which was displayed in the arterial phase (red square).(b) Disappearance of enhancement and reduction of the HCC was displayed on CECT after the procedure (red square). (c) A small nodule was first detected in the lower lobe of the right lung (square) on CT after the procedure. Partial lung resection was performed, and it was histologically diagnosed as inflammatory granuloma.

Two months after the procedure, complete necrosis of the S3 and S5 tumors was displayed on contrast-enhanced CT (CECT); however, a small nodule was found in the right lower lobe of the lung, which was suspected to be a metastasis (Figure 3). Partial lung resection under video-assisted thoracic surgery was performed and it was pathologically diagnosed as an inflammatory granuloma rather than a malignant tumor. LDLT was performed 12 months after DEM-TACE.

## Pathological findings

### Underlying liver disease

**Figure 4.**
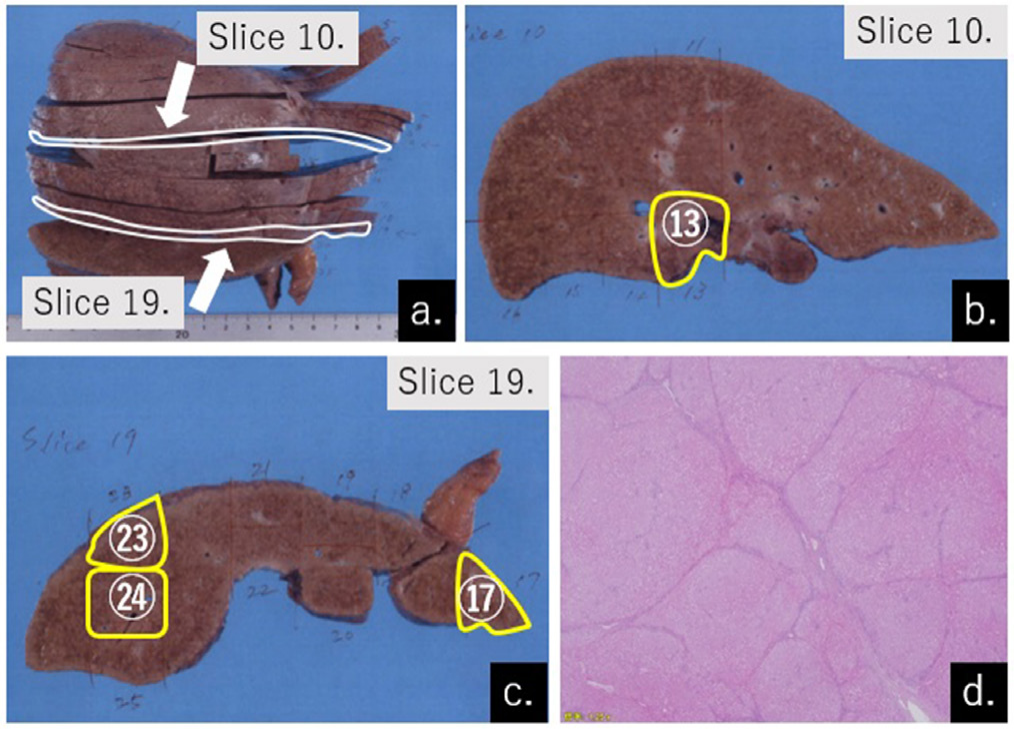
Gross appearance and cut specimens of the liver demonstrating underlying liver disease (a, b, c) Rough and small granules were observed on the entire surface of the liver. Formation of regenerative nodules of 3 mm or less was observed on the sliced surfaces.(a) The resected liver was cut every 3 mm, and no HCC nodules were observed on the surface of the cut liver. Slices no. 10 and 19 were selected, and specimen slides were made from areas no. 13 (b), and 17, 23, and 24 (c) for pathological observation.(b) One HepaSphere particle was detected in area no. 13 from slice no. 10. (c) 5, 3, and 13 HepaSphere particles were detected in areas no. 17, 23, and 24, from slice no. 19. (d) Each pseudolobule was separated by thin bridging fibrosis, mostly portal–portal bridging. This indicated advanced cirrhosis of the liver (hematoxylin and eosin stain, low-power field).

The total weight of the resected liver was 880 g in Figure 4. Rough and small granules were observed on the entire surface of the liver. Formation of regenerative nodules of 3 mm or less were observed on the cut surface. Each pseudolobule was separated by thin bridging fibrosis, mostly portal-portal bridging, indicating advanced liver cirrhosis. The dilation of Glisson’s sheath and lymphocyte infiltration were mild.

### HCC and HepaSphere

**Figure 5.**
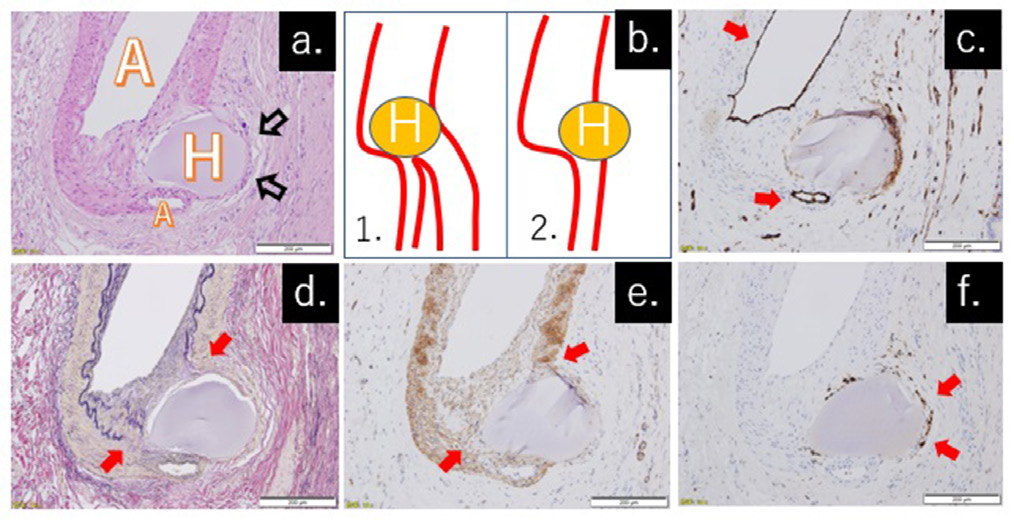
(a) Detection of the noted HepaSphere particle on hematoxylin and eosin staining. One artery (A) ran from the top to the bottom of the image and a HepaSphere particle (H) was observed near the artery. There was no arterial wall on the right side of the particle, but another group of cells was observed (arrows). (b) The 2 possible hypotheses of the results in Figure 5a. (1) The HepaSphere particle was located at an artery branch site, and (2) the HepaSphere particle leaked out from the artery. (c) The vascular endothelium was stained with a CD31 antibody. On the right side of the HepaSphere particle, there was no arterial endothelium. (d) EVG staining showed staining of the elastic fibers of the tunica media. (e) SMA staining showed staining of the smooth muscle cells of the tunica media, indicating that the right side of the HepaSphere particle was not covered by the tunica media. (f) CD68 staining showing staining of histiocytes. The group of cells on the right side of the HepaSphere particle were giant multinucleated cells, which confirmed that the right side of the particle was the interstitial side. Therefore, the immunostaining results confirmed that the second hypothesis is correct.

In the specimens cut into 3 mm slices, neither HCC tumors nor necrotic nodules remained in the S3 and S5. No cancerous cells were detected histologically in the area where the tumor was considered to be present, and 22 HepaSphere particles were detected. The size (largest and smallest diameter) and deformation of the final HepaSphere were 241.6 ± 52.5 µm, 186.5 ± 41.4 µm, and 22.6 ± 13.0%, respectively (Table 1). All HepaSphere particles (*n* = 22) were localized extravascularly in the interstitial space, and foreign body giant cells were gathered around them. No apparent inflammatory cell migration was observed. HepaSphere particle was observed near an artery (Figure 5). In Figure 5a-f, 2 hypotheses could be considered, namely, that the HepaSphere particle simply exists at the location where the artery branches into 2 (Figure 5b-1) or that the HepaSphere particle was eliminated from the artery to the interstitial space (Figure 5b-2). It is difficult to distinguish by hematoxylin and eosin staining alone whether the group of dark pink cells seen on the right side of the HepaSphere particle is the vascular endothelium or a group of cells present in the interstitial space. Therefore, immunostaining using other antibodies were also performed. On CD31 staining, vascular endothelial cells of the arteries further upstream and downstream were stained a dark brown color and no endothelium was present on the right side of the HepaSphere (Figure 5c). On EVG and SMA staining, the elastic layer (purple color) and smooth muscle cells (light brown color) were torn between the HepaSphere and the arterial wall (Figure 5d,e). On CD68 staining, in which histocytes were stained a dark brown, a group of cells on the right side of the HepaSphere particle was stained and was confirmed to be giant multinucleated cells (Figure 5f). Thus, the transvascular migration of HepaSphere was clearly confirmed by immunostaining.

**Table 1.** Comparison with previous studies regarding final size, deformation, and location of HepaSphere particles (50–100 µm) after embolization

Study	Species/ organ	Time after embolization	No. of particles	Largest diameter (µm)	Deformation (%)	Location
Luis et al^1^	Pig/ kidney	4 weeks	608	230.2 ± 62.5	17.1 ± 12.3	EV^*a*^
Bilbao et al.^4^	Pig/ kidney	48 hours / 4 weeks	428	225.3 ± 67	26 ± 19.7	IV / EV
Present case	Human/ liver	12 months	22	241.6 ± 52.5	22.6 ± 13.0	EV

IV, intravascular; EV, extravascular;

a Exact location unclear.

## Discussion

HepaSphere, superabsorbent polymer microsphere, is one of the calibrated, nondegradable embolic agents. It has used for the trans-arterial embolotherapy of hypervascular tumor. Especially DEM-TACE for HCC has become widespread.^5,6^ Several reports have been published on the use of HepaSphere *in vivo*, including pathological analyses.^1,4^ The largest diameter, and deformation of HepaSphere in the present case were equivalent to those in previous reports in which embolization with HepaSphere (50–100 µm) was performed in porcine renal arteries (Table 1). Recently, some authors reported that DEM-TACE with new and smaller HepaSphere particles (30–60 µm) was effective and safe.^7^ However, in Japan, this smaller type of HepaSphere is not commercially available. Therefore, we used the reduced expansion technique. Sato et al reported that HepaSphere particles (50–100 µm) swelled to a mean diameter of 188.4 µm when using this technique, compared to 404.9 µm when using the normal technique.^3^ Although this can be thought of as an ideal size, the artery diameter that can actually be embolized by the HepaSphere remains unclear. In the present case, the artery diameter that was embolized by HepaSphere could not be calculated because all HepaSphere particles leaked out of the arteries on long-term follow up.

There are 2 important and interesting points in the present case. Firstly, all HepaSphere microspheres leaked out of the arteries. In 1988, Tomashefski et al first reported transvascular migration of the embolic material polyvinyl alcohol (PVA), and its mechanism.^8^ The anticipated mechanism consists of the following 4 steps: 1) formation of a thrombus in the blood vessel, 2) early tissue destruction of the inner elastic plate and inflammation of the blood vessel wall, 3) complete destruction of the vessel wall, and 4) leakage of the embolic material out of the blood vessel, and replacement of the vessel wall with fibrous tissue. Laurent et al compared embolization with PVA (600–1,000 µm) and EmboSphere (700–900 µm) in uterine sheep arteries.^9^ Regarding PVA, 99.2% of the particles was present in the arteries, whereas for EmboSphere, 54.4% was present in the arteries, and 45.6% leaked out into the extravascular interstitial space. Noncalibrated PVA frequently aggregated and became trapped in arteries that were larger than the actual size of the PVA particles, whereas EmboSphere seldomly aggregated, and only embolized arteries of the expected diameters. Therefore, smaller and calibrated microspheres may reach more distal arteries and lead to more extravascular leakage. In the present case, the largest HepaSphere diameter was 241.6 ± 52.5 µm. The diameter is expected to be about 200 µm or less when the reduced expansion technique is used. In any case, it was speculated that the calibrated HepaSphere particles, which are much smaller than EmboSphere particles (700–900 µm), underwent extravascular migration after reaching the periphery. The presence or absence of aggregation in our present case remains unknown, as short-term pathological follow-up was not performed.

Second, to our knowledge, this is the first study to detect the extravascular migration of HepaSphere particles by immunostaining. The extravascular migration of embolic material has been reported for both PVA and EmboSphere.^8,9^ That is, histopathological images of the embolic particles breaking the blood vessel wall and flowing out of the blood vessel were obtained. The histopathological detection of HepaSphere in the interstitium has also been reported.^1,4,10^ Although it is expected that a similar phenomenon will occur in the blood vessels, no clear histopathological images of the HepaSphere particles actually rupturing the blood vessel wall and going out of the artery had previously been obtained. In the present study, extravascular migration of HepaSphere was clearly confirmed by immunostaining.

The next problem was regarding the timing at which transvascular migration occurred. In DEM-TACE for primary breast cancer, which we performed previously, extravascular migration was already observed 2–5 months after the procedure.^11^ Hori et al reported that extravascular leakage was already observed at 4 weeks after the procedure.^10^ Bilbao et al reported that extravascular leakage was not observed at 48 h but was observed at 4 weeks after the procedure.^4^ Therefore, HepaSphere was predicted to leak out at a relatively early stage (within 48 h to 4 weeks). Although in our present study we performed long-term follow-up (at 1 year after embolization), we observed histopathological images of HepaSphere particles destroying elastic fibers and coming out of the blood vessels, suggesting that the extravascular migration phenomenon remained incomplete for some reason. It has been reported that the diameter of arteries that can be embolized by HepaSphere can be predicted, similarly to other spherical embolic material. In the present case the transvascular migration that was observed indicated that peripheral arteries can be embolized by HepaSphere.

## Conclusion

All the HepaSpheres detected in the examined pathological specimen were noted to be extravascular.

## Learning points

1. In long-term follow up of DEM-TACE using HepaSphere, all HepaSphere particles were found in the stroma and this was the first histological demonstration of extravascular migration.
2. The extravascular migration findings suggest that the embolic agent reached distal parts of the artery.
